# MacP bypass variants of *Streptococcus pneumoniae* PBP2a suggest a conserved mechanism for the activation of bifunctional cell wall synthases

**DOI:** 10.1128/mbio.02390-23

**Published:** 2023-10-17

**Authors:** Caroline Midonet, Sean Bisset, Irina Shlosman, Felipe Cava, David Z. Rudner, Thomas G. Bernhardt

**Affiliations:** 1 Department of Microbiology, Harvard Medical School, Blavatnik Institute, Boston, Massachusetts, USA; 2 Department of Molecular Biology, Umeå University, Umeå, Sweden; 3 Department of Biological Chemistry and Molecular Pharmacology, Harvard Medical School, Blavatnik Institute, Boston, Massachusetts, USA; 4 Laboratory for Molecular Infection Medicine Sweden (MIMS), Umeå Center for Microbial Research (UCMR), Umea, Sweden; 5 Department of Molecular Biology, Science for Life Laboratory (SciLifeLab), Umeå University, Umeå, Sweden; 6 Howard Hughes Medical Institute, Chevy Chase, Maryland, USA; Universite de Geneve, Genève, Switzerland

**Keywords:** penicillin-binding proteins, peptidoglycan, cell envelope, cell wall

## Abstract

The peptidoglycan (PG) layer protects bacteria from osmotic lysis and defines their shape. The class A penicillin-binding proteins (aPBPs) are PG synthases that possess both glycan polymerization and crosslinking activities needed for PG biogenesis. In Gram-negative bacteria, aPBPs require activation by outer membrane lipoproteins, which are thought to stimulate their cognate synthase by inducing conformational changes that promote polymerase function. How aPBPs are controlled in Gram-positive bacteria is less clear. One of the few known regulators is MacP in *Streptococcus pneumoniae* (*Sp*). MacP is required for the activity of *Sp* PBP2a, but its mode of action has been obscure. We therefore selected for PBP2a variants capable of functioning in the absence of MacP. Amino acid substitutions that bypassed the MacP requirement for PBP2a function *in vivo* also activated its polymerase activity *in vitro*. Many of these changes mapped to the interface between the transmembrane (TM) helix and polymerase domain in a model PBP2a structure. This region is conformationally flexible in the experimentally determined structures of aPBPs and undergoes a structural transition upon binding the substrate-mimicking drug moenomycin. Our findings suggest that MacP promotes PG polymerization by altering the TM-polymerase domain interface in PBP2a and that this mechanism for aPBP activation may be broadly conserved. Furthermore, *Sp* cells expressing an activated PBP2a variant displayed heterogeneous shapes, highlighting the importance of proper aPBP regulation in cell morphogenesis.

**IMPORTANCE:**

Class A penicillin-binding proteins (aPBPs) play critical roles in bacterial cell wall biogenesis. As the targets of penicillin, they are among the most important drug targets in history. Although the biochemical activities of these enzymes have been well studied, little is known about how they are regulated in cells to control when and where peptidoglycan is made. In this report, we isolate variants of the *Streptococcus pneumoniae* enzyme PBP2a that function in cells without MacP, a partner normally required for its activity. The amino acid substitutions activate the cell wall synthase activity of PBP2a, and their location in a model structure suggests an activation mechanism for this enzyme that is shared with aPBPs from distantly related organisms with distinct activators.

## INTRODUCTION

The peptidoglycan (PG) cell wall surrounds most bacterial cells, providing their characteristic shape and protecting them from osmotic lysis. This essential layer of the cell envelope is made from glycan strands composed of repeating units of N-acetylglucosamine (GlcNAc) and N-acetylmuramic (MurNAc) acid (1). A short peptide is attached to the MurNAc sugar and is used to form crosslinks between adjacent glycans, generating a polymeric matrix that encapsulates and fortifies the cytoplasmic membrane.

There are two families of synthases that build the PG layer. The class A penicillin-binding proteins (aPBPs) are one type. They are bifunctional enzymes possessing both PG glycosyltransferase (PGTase)/polymerase activity for glycan polymerization and transpeptidase (TPase) activity for peptide crosslinking. Complexes between SEDS family proteins and class B penicillin-binding proteins (bPBPs) make up the second major type of PG synthase. In this case, the SEDS protein provides the PG polymerase activity, and the bPBP performs the crosslinking reaction (2–4). The SEDS-bPBP complex FtsW-FtsI [FtsW-PBP2x in *Streptococcus pneumoniae* (*Sp*)] forms the essential PG synthase of the cell division machinery, and in rod-shaped organisms, the related RodA-PBP2 (RodA-PBP2b in *Sp*) complex provides the PG synthesis activity of the Rod system (elongasome) needed for cell elongation and shape maintenance (5–9). The aPBPs are thought to maintain cell wall integrity by fortifying a foundational, ordered PG network produced by SEDS-bPBP complexes, which function within morphogenic machines (4). Evidence suggests that the aPBPs may also function in the repair of damaged areas of the PG matrix (4, 10–12). The activities of both types of PG synthases must be properly regulated and balanced to prevent aberrant morphogenesis and the many detrimental consequences of an altered cell wall structure. The focus of this report is the regulation of the aPBPs.

In Gram-negative bacteria, the aPBPs require activation by outer membrane lipoproteins. In the model organism *Escherichia coli*, the two major aPBPs are PBP1a and PBP1b. They are activated by the unrelated lipoproteins LpoA and LpoB, respectively (11, 13). These activators each interface with their cognate synthase through a regulatory domain located in the extracytoplasmic region of the enzymes that also includes the conserved PGTase and TPase domains (14). Like the lipoproteins, the two aPBP regulatory domains, OB/ODD for PBP1a and UB2H for PBP1b, are unrelated in sequence or structure (11, 13, 14). When the lipoproteins bind to their cognate regulatory domain, they are thought to induce conformational changes in the target aPBP that activate their ability to polymerize PG (15–17). Although TPase activity may also be affected, it is the stimulation of glycan strand production that is likely to be the key regulatory step given that it is the first enzymatic reaction of PG synthesis and is responsible for producing the substrate for crosslinking by the TPase (18).

By contrast with Gram-negative bacteria, much less is known about aPBP regulation in Gram-positive organisms. In this regard, the ovoid-shaped pathogen *Streptococcus pneumoniae* (*Sp*) has been a useful model. Like *E. coli*, it has two aPBPs that form a synthetically lethal pair (19–21). Mutants inactivated for either PBP1a or PBP2a are viable, but the simultaneous inactivation of both enzymes is lethal (22). We previously took advantage of this genetic property to screen for potential regulators of these aPBPs (23). CozE was found to be an essential protein that became dispensable in the absence of PBP1a (23). It was subsequently shown to be required for the localization of PBP1a to midcell, thereby preventing lethal shape defects induced by spurious, delocalized PG synthesis around the cell periphery. MacP was another aPBP regulator identified in *Sp* based on its synthetic lethal relationship with PBP1a (24). Inactivation of MacP is lethal in cells lacking PBP1a, suggesting it is required for PBP2a function (24). Accordingly, MacP was shown to interact with PBP2a in *Sp* cells. Additionally, MacP was found to be a substrate for the kinase StkP, a global cell-cycle regulator (25, 26), and a phosphoablative MacP variant was shown to be defective in its ability to promote PBP2a activity without affecting its interaction with the synthase (24). MacP is a 104 amino acid protein with an N-terminal globular domain in the cytoplasm and a predicted C-terminal transmembrane helix. Only a few amino acids of the MacP C-terminal domain are likely to extend into the extracytoplasmic space where the enzymatic domains of PBP2a reside. It is, therefore, unclear how MacP might promote PBP2a activity or how the phosphorylation of its N-terminal cytoplasmic domain might modulate its function (24).

The isolation and characterization of activator bypass variants of *E. coli* aPBPs that function without their cognate lipoprotein partner has been a fruitful strategy to investigate the mechanism of aPBP regulation in Gram-negative bacteria (13, 17, 27). We therefore extended this approach to investigate the role of MacP in promoting PBP2a function in *Sp* cells and better understand how aPBP activity is controlled in Gram-positive bacteria. PBP2a variants capable of functioning in the absence of MacP were selected. Several of the amino acid substitutions mapped to the interface between the transmembrane (TM) helix and polymerase domain in a model PBP2a structure. This region in the structures of aPBPs from *Escherichia coli* and *Staphylococcus aureus* is conformationally flexible and undergoes a structural transition upon binding the substrate-mimicking drug moenomycin (28–30). AlphaFold modeling also indicates that this region is the likely interaction site for the small extracytoplasmic C-terminal peptide of MacP (31, 32). Our findings therefore suggest that MacP promotes PG polymerization by altering the TM-polymerase domain interface in PBP2a and that this mechanism for aPBP activation may be broadly conserved. Finally, *Sp* cells expressing an activated PBP2a variant displayed heterogeneous shapes, highlighting the importance of proper aPBP regulation in cell morphogenesis.

## RESULTS

### Selection for PBP2a variants that function in the absence of MacP

To identify PBP2a variants that bypass the MacP requirement for function, the *pbp2a* gene was mutagenized by error-prone PCR and inserted into a plasmid such that it was adjacent to a chloramphenicol resistance cassette (Cam^R^) and flanked by regions of upstream and downstream homology to the native *pbp2a* locus. The plasmid library was transformed into an *Sp* strain (SP1047) deleted for *pbp1a*, *pbp2a*, and *macP* with an ectopic copy of *pbp1a* under the control of a Zn^2+^-regulated promoter (P*_Zn_-pbp1a*). Due to the synthetic lethal relationship between PBP1a and PBP2a/MacP, this strain is only viable in the presence of added Zn^2+^ to induce PBP1a production. Therefore, a library of recombinants where the plasmid-borne *pbp2a* gene replaced the deletion allele was initially generated by selection on agar containing ZnCl_2_ (200 µM) and chloramphenicol. A mock library was also generated in which an unmutagenized version of the *pbp2a* plasmid was similarly transformed into the SP1047 selection strain. The mutagenized and mock libraries were plated on medium with ZnCl_2_ to estimate the total number of transformants and on medium lacking ZnCl_2_ to deplete PBP1a. Under the no-ZnCl_2_ condition, cells rely on PBP2a activity for growth, but because the strain also lacks MacP, PBP2a variants capable of functioning in the absence of MacP are required for viability. Following selection, the plating efficiency of the mutagenized *pbp2a* library (3.2 × 10^−5^) was 10 times higher than that of the mock library (3.0 × 10^−6^), indicating that the majority of survivors from the mutagenized library were likely to contain gain-of-function mutations in *pbp2a* that promote growth of the *∆macP* cells depleted for PBP1a (Fig S1).

To confirm that the MacP bypass phenotype was linked to *pbp2a*, the *pbp2a* locus from the survivors was back-crossed into the SP1047 (*∆pbp1a ∆macP ∆pbp2a P_Zn_-pbp1a*) selection strain. Fifteen mutants capable of growing in the absence of ZnCl_2_ were confirmed, and their *pbp2a* locus was sequenced to identify potential changes. All the sequenced *loci* contained mutations in the PBP2a coding region, with many possessing multiple base changes (Table S1).

Seven of the mutants encoded PBP2a variants with an A77T substitution either alone (3/7) or with an additional P357S (4/7) change. Given the prevalence of the A77T variant among the isolates, we focused on characterizing its activity further.

### PBP2a(A77T) bypasses the MacP requirement for synthase function

**Fig 1:**
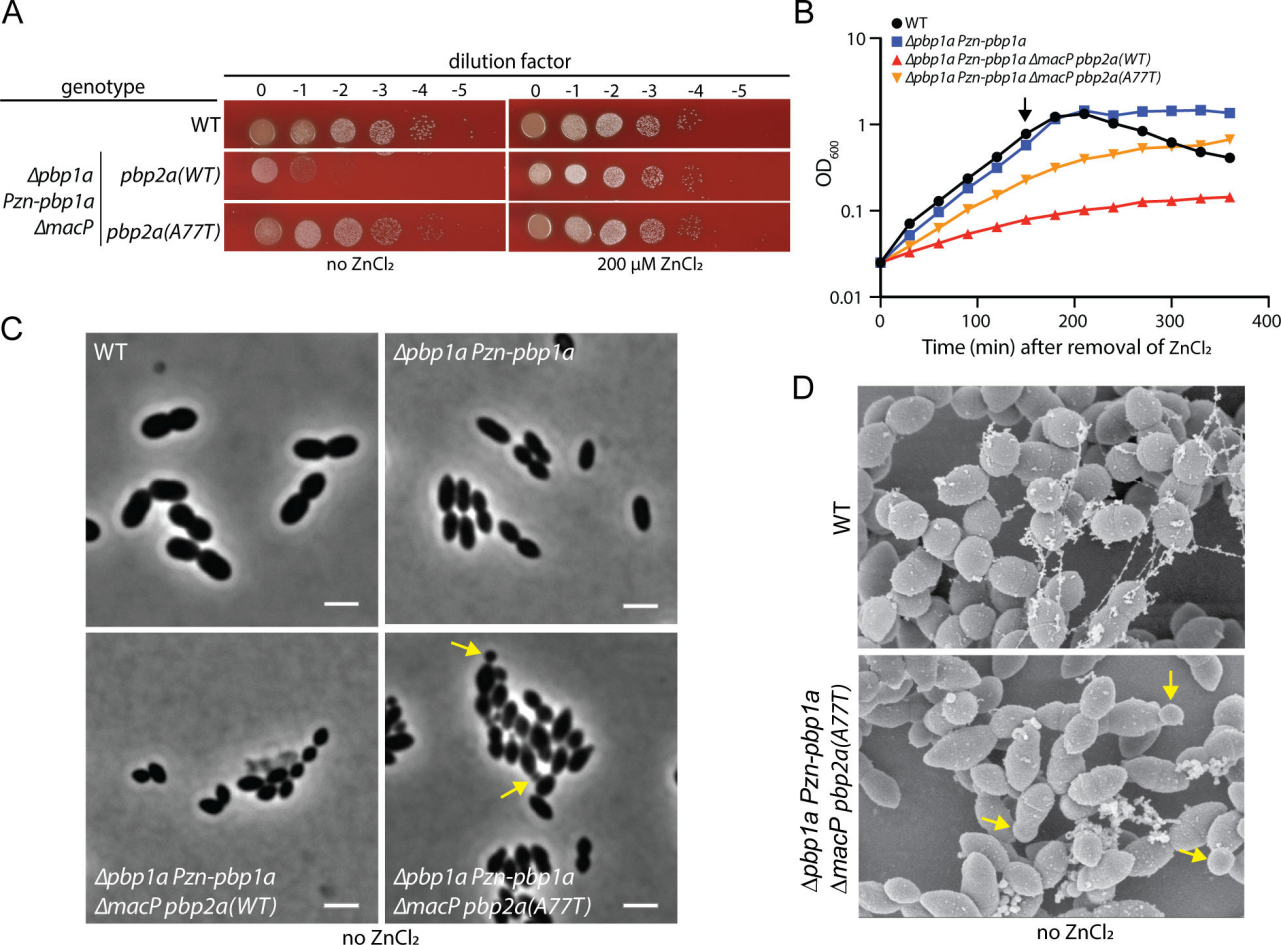
PBP2a(A77T) bypasses the requirement for MacP. (**A**) Viability assays of the indicated strains on blood agar plates in the absence and presence of 200 µM ZnCl_2_. Cells harboring PBP2a(A77T) are viable in the absence of MacP and PBP1a. (**B**) Representative growth curves of the indicated strains. ZnCl_2_ was removed at 0 min. The black arrow indicates the time point at which cells were analyzed by phase contrast microscopy. (**C**) Representative phase contrast images of strains from (**B**) after 150 min of growth in the absence of ZnCl_2_. Minicells are highlighted with yellow arrows. Scale bar, 2 µm. (**D**) Scanning electron micrographs (magnification 15,000x) of the indicated strains grown for 150 min in the absence of ZnCl_2_. Yellow arrows highlight mini cells. The data in Fig. 1A through C are representative of at least three biological replicates. The micrographs in Fig. 1D are from one biological replicate.

As expected from previous work, *Sp* cells encoding wild-type (WT) PBP2a that also lacked MacP displayed a severe plating defect on blood agar medium when PBP1a was depleted (Fig. 1A) (24). However, in the presence of PBP2a(A77T), cells deleted for *macP* plated relatively efficiently upon PBP1a depletion (Fig. 1A). Importantly, deletion of *pbp1a* was possible in *∆macP* cells harboring *pbp2a(A77T)* but not *pbp2a(WT)*, indicating that leaky production of PBP1a from the *P_Zn_-pbp1a* allele is not required for PBP2a(A77T) to promote growth in the absence of MacP when PBP1a is depleted (Fig. S1).

In liquid medium, PBP1a depletion in otherwise wild-type cells does not cause an observable growth phenotype except for a reduced propensity for culture autolysis in the stationary phase (Fig. 1B). However, as observed previously, PBP1a depletion resulted in smaller cells relative to the wild type (Fig. 1C). Growth in liquid medium was greatly reduced upon PBP1a depletion in cells lacking MacP (Fig. 1B). Consistent with prior results, the cell size defect was more pronounced in this background, and lysed cells were commonly observed in the culture (Fig. 1C) (24). As with growth on agar, the PBP2a(A77T) variant largely rescued the growth defect of PBP1a depletion in *∆macP* cells, but growth was still impaired relative to cells depleted for PBP1a that retained wild-type PBP2a and MacP (Fig. 1B). Notably, many of the cells relying on PBP2a(A77T) function in the absence of MacP displayed an aberrant morphology in which small “minicells” were attached to relatively normal ovoid cell bodies (Fig. 1C and D). Whether the observed minicells form as a result of misplaced division events or problems with symmetric elongation of the cell halves remains unclear. Nevertheless, the morphological defects indicate that despite its ability to promote growth in the absence of MacP and PBP1a, PBP2a(A77T) is not sufficient to maintain normal cell shape.

### Identification of additional PBP2a variants that function without MacP

**Fig 2:**
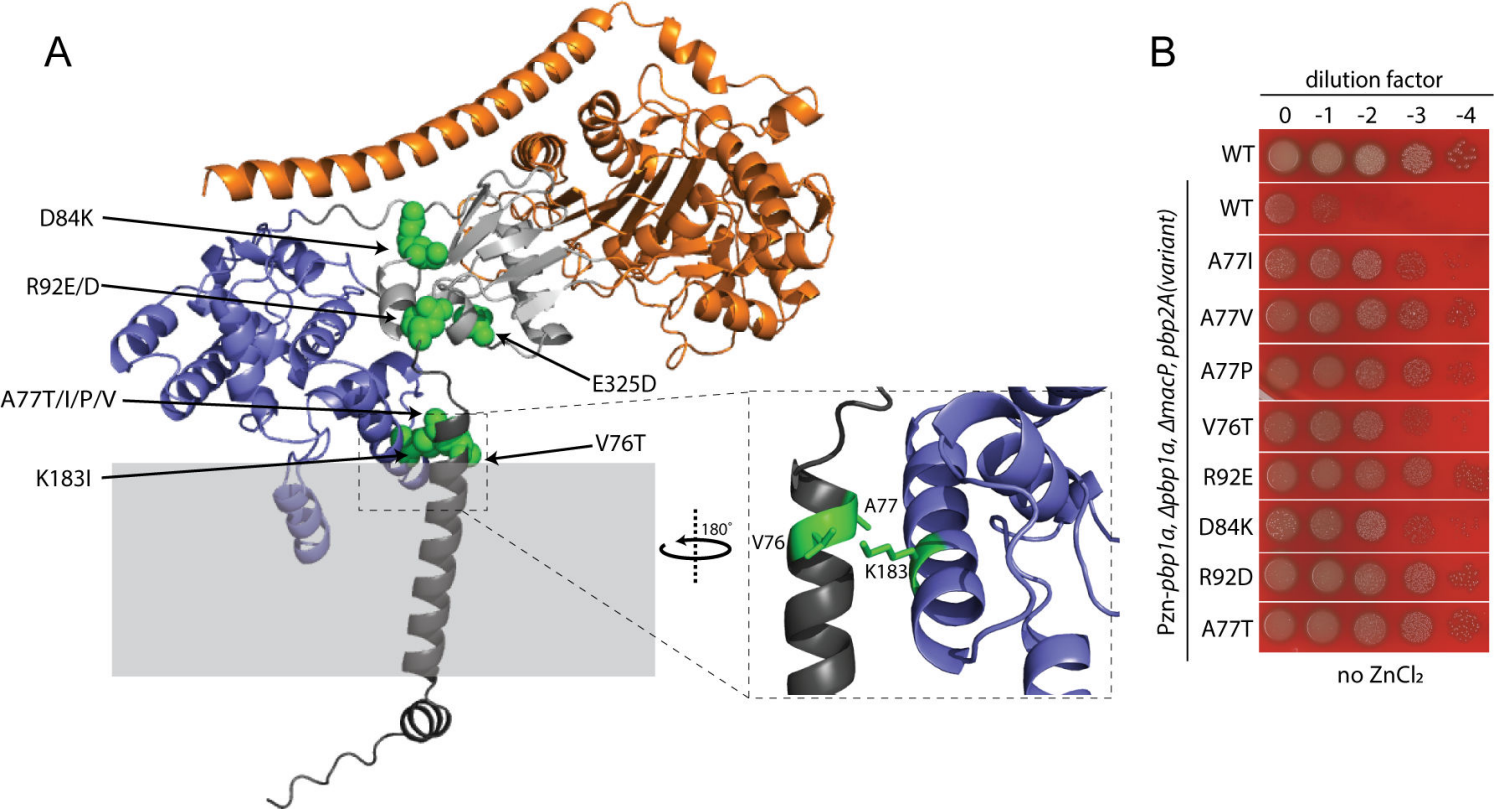
Amino acid substitutions in the PGTase/TM interface and linker in PBP2a variants that bypass MacP. (**A**) AlphaFold model of PBP2a with highlighted amino acids (green) that, when substituted, can bypass the requirement for MacP. The predicted structure is colored as follows: cytoplasmic and transmembrane segment (dark-gray), linker between PGTase and TPase domain (light-gray), PGTase domain (blue), and TPase domain and C-terminal helix (orange). A zoom in on the interface between the TM segment (dark-gray) and alpha helix 6 of the glycosyltransferase (GT) domain is shown in the dashed rectangle. Substitutions in the green residues conferred a MacP-bypass phenotype. (**B**) Spot dilutions of the indicated strains on blood agar plates in the absence of ZnCl_2_. All the PBP2a mutants sustain growth in the absence of MacP and PBP1a. The data in Fig. 2B are from one of three biological replicates.

The A77T substitution is predicted to lie at the interface between the transmembrane and PGTase domains in a high-confidence model structure of PBP2a (Fig. 2A; Fig. S2B). Notably, other MacP bypass variants with amino acid substations in this region (L73S and K183I) were also isolated in the original selection, but these isolates also contained several additional substitutions (Fig. 2A; Table S1). To determine if additional changes at residue A77 or in its vicinity can similarly bypass the requirement for MacP, we generated 37 new PBP2a variants with amino acid substitutions in the region between amino acids 75 and 101 (Fig S2A). We also separately tested E325D and K183I substitutions that were both identified in our selection. The latter variant was generated without the additional changes found in the original mutant isolate. Variants were introduced into the SP1047 (*∆pbp1a ∆macP ∆pbp2a P_Zn_-pbp1a*) selection strain, replacing the *∆pbp2a* allele. The strains were then tested for growth in the absence of ZnCl_2_ to determine whether the PBP2a variants could bypass the requirement for MacP. In addition to the A77T substitution, changes in this position from Ala to Ile, Pro, and Val resulted in a MacP bypass phenotype (Fig. 2B; Fig. S2A). Other changes that promoted PBP2a function in the absence of MacP were as follows: V76T, D84K, R92D, R92E, K183I, and E325D. (Fig. 2B; Fig. S2A and C). In the model structure, these changes mapped to the TM-PGTase domain interface or the linker domain that connects the PGTase and TPase enzymatic domains (Fig. 2A).

### The A77T and D84K substitutions activate the polymerase activity of PBP2a

**Fig 3:**
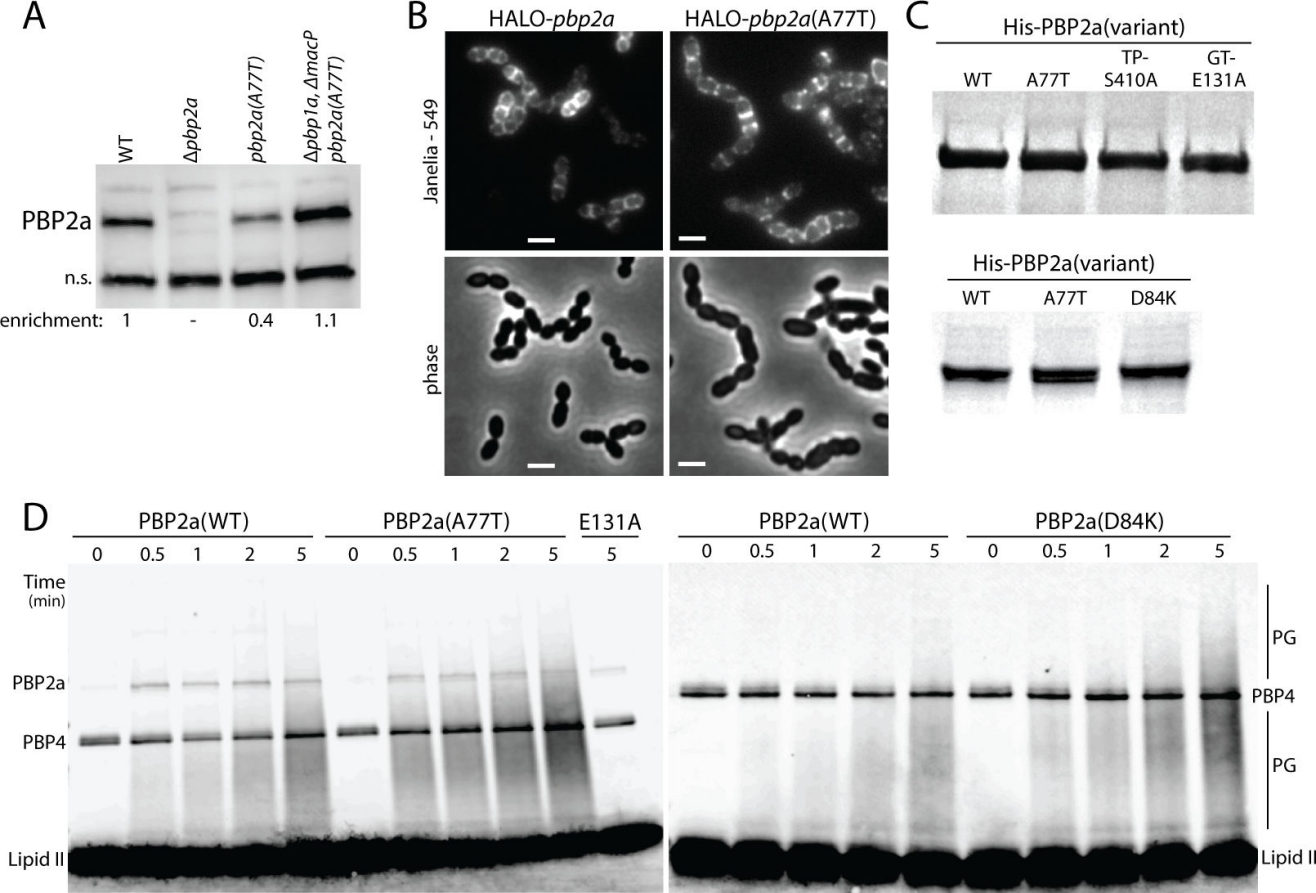
PBP2a bypass mutants have increased glycosyltransferase activity. (**A**) Representative immunoblot of PBP2a in the indicated strains. A non-specific (ns) protein that was recognized by the anti-PBP2a antibody was used to control for loading. The levels of PBP2a relative to the wild type are indicated below the blot. (**B**) Representative images of fluorescence and phase contrast microscopy of strains harboring HALO-PBP2a or HALO-PBP2a(A77T). Scale bar, 2 µm. (**C**) InstantBlue-stained gels of the purified His-PBP2a variants used in the glycosyltransferase assays. The transpeptidase and glycosyltransferase catalytic mutants, His-PBP2a(S410A) and His-PBP2a(E131A), are labeled TP- and GT-, respectively. (**D**) Representative blot of glycan strand polymerization assay using purified proteins and lipid II. Time points (in min) are indicated above the blot. The reaction products and remaining substrate were labeled with biotinylated D-lysine using *S. aureus* PBP4, resolved by SDS-PAGE, transferred to PVDF (polyvinylidene fluoride), and detected by IRDye 800CW Streptavidin. Free lipid II and glycan strands (PG) are indicated. Some PBP4 and PBP2a become biotinylated in the labeling reaction and are detected by IRDye 800CW Streptavidin. All data in this figure are from one of three biological replicates.

Immunoblotting indicated that the levels of PBP2a(A77T) were not elevated relative to PBP2a(WT) in an otherwise wild-type background or a strain deleted for both *macP* and *pbp1a* where the PBP2a(A77T) variant is required for viability (Fig. 3A; Fig. S3A). Thus, the bypass phenotype does not result from an increased accumulation of the PBP2a protein. To determine if the A77T substitution alters the subcellular localization of PBP2a, we generated functional fusions to the HALO tag, which can be detected and tracked by fluorescence microscopy following labeling with the dye JF-549 (Fig. 3B; Fig. S3B). Both the HALO-PBP2a(WT) and HALO-PBP2a(A77T) fusions were produced at comparable levels (Fig. S3C) and displayed similar localization patterns with the signal dispersed around the cell periphery as well as enrichment at the septa of some cells (Fig. 3B). Therefore, the MacP bypass phenotype does not appear to stem from altered protein localization.

To test the effect of substitutions at the TM-PGTase domain interface or near the linker domain on the activity of PBP2a, 6xHis-tagged fusions of WT as well as the A77T and D84K variants were overexpressed in *Escherichia coli* and purified by Ni^2+^-NTA chromatography (Fig. 3C). We also tried overexpressing and purifying variants with changes at position 92, but the yield of quality protein was poor for unknown reasons. Variants with a substitution in the PGTase (E131A) or TPase (S410A) catalytic sites that inactivate polymerase or crosslinking activity, respectively, were also purified as controls (Fig. 3C). The PGTase activity of each protein was then assessed in reactions with lipid II substrate purified from *Enterococcus faecalis* as described previously (5, 33–35). The reactions were terminated at various times after initiation, and glycan polymers were labeled via the addition of *Staphylococcus aureus* PBP4 and biotin-D-Lys, which results in the addition of the biotinylated amino acid to the PG stem peptide. The labeled polymers were then separated by SDS-PAGE, transferred to a membrane, and detected with fluorescently labeled streptavidin. PBP2a(WT) produced an increasing amount of glycan polymers that grew in length over the time course (Fig. 3D). No activity was observed for PBP2a(E131A) as expected for a PGTase-defective variant (Fig. 3D; Fig. S3E), indicating the absence of contaminating *E. coli* aPBP activity. Additionally, we did not observe a difference between the activity of PBP2a(WT) and the TPase-defective variant PBP2a(S410A) (Fig. S3E). Thus, PBP2a does not appear to be capable of crosslinking the *E. faecalis* lipid II substrate. The SDS-PAGE and blotting assays are therefore reporting on the PGTase activity of the enzyme. Importantly, PBP2a(A77T) and PBP2a(D84K) produced PG polymers at an enhanced rate relative to PBP2a(WT), with the activation of the PBP2a(A77T) variant being the most pronounced (Fig. 3D). We therefore conclude that these substitutions activate the PGTase function of the enzyme and that this activation is likely responsible for their ability to bypass the MacP requirement for function *in vivo*.

### Hyperactive PBP2a alters cell wall composition and cell morphology

**Fig 4:**
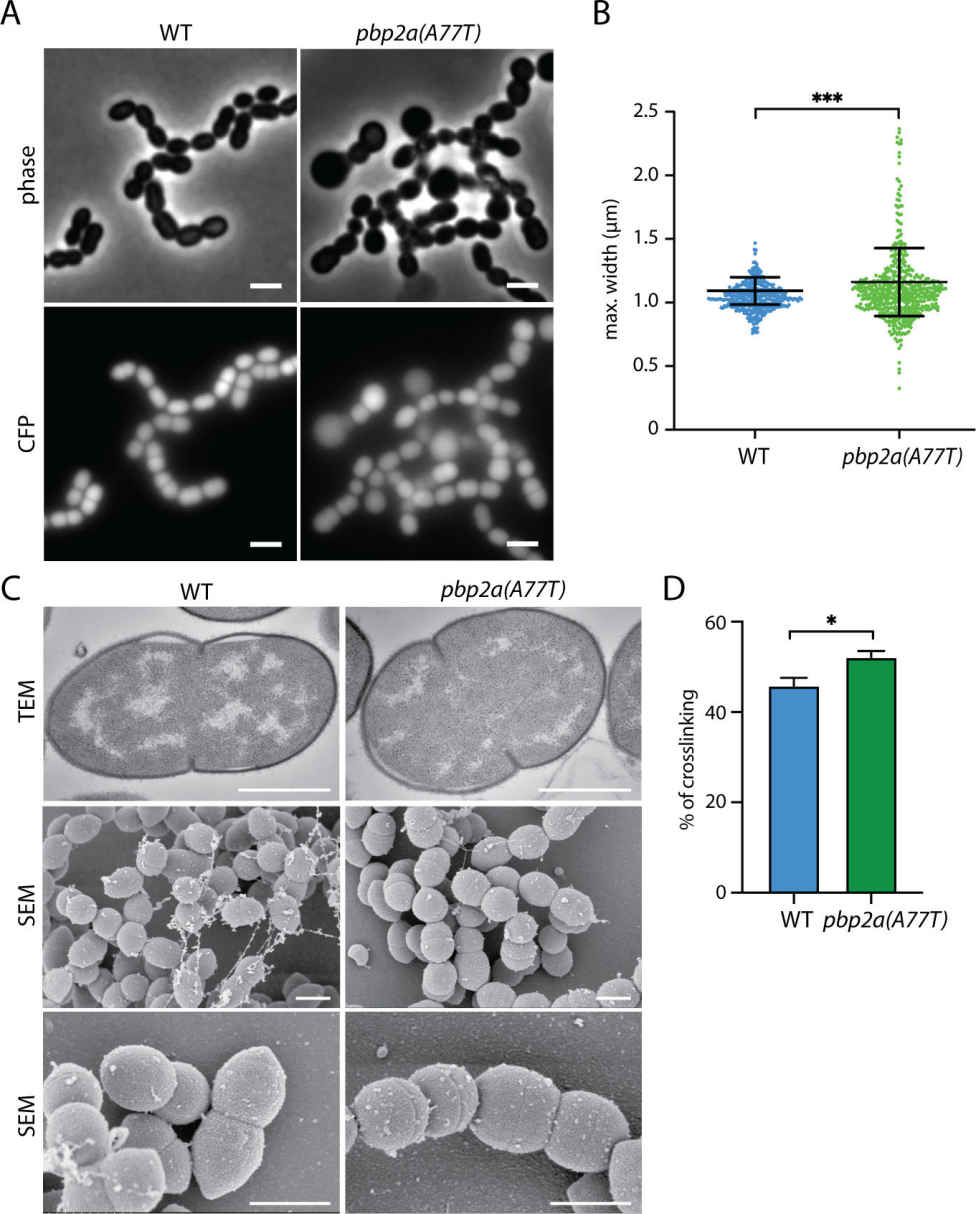
Cells harboring PBP2a(A77T) have aberrant morphologies and changes in PG crosslinking. (**A**) Representative fluorescence and phase contrast images of the indicated strains. Both strains constitutively express cytoplasmic CFP. Scale bar 2 µm. (**B**) Representative plots of cell width distributions in WT (*n* = 380) and pbp2A(A77T) (*n* = 509). The difference between the two strains is statistically significant (***) with *P* = 0.0003 (non-parametric *t*-test). (**C**) Transmission electron microscopy and scanning electron microscopy of the indicated strains. TEM magnification is 40,000. Scale bar, 1 µm. SEM magnification is 15,000x (middle) and 35,000x (bottom). Note that the middle image from wild-type cells is a duplicate of the SEM image from Fig. 1D. It is presented here to more easily compare cell morphology with that of the PBP2a(A77T)-producing strain. (**D**) Bar graph shows the percentage of cell wall crosslinking in the indicated strains. The difference is statistically significant (*) with *P* = 0.024. The data in panels A, B, and D, are from one of three biological replicates. The SEM and TEM are from one biological replicate.

We next wished to determine the consequence of having hyperactivated PBP2a in cells with an otherwise normal complement of PG synthesis factors. Therefore, the *pbp2a(A77T)* allele was transferred to our wild-type strain background. Although there was not a strong growth defect caused by the presence of the PBP2a(A77T) variant (Fig. S4A), the altered enzyme caused significant morphological heterogeneity (Fig. 4). Cells expressing either PBP2a(WT) or PBP2a(A77T) were engineered to produce a cytoplasmic CFP (cyan fluorescent protein) marker for image segmentation and quantitative analysis of cell shape (Fig. 4A). Compared with the wild type, many cells producing PBP2a(A77T) were much larger and wider than normal. Image analysis confirmed the broader distribution of cell widths in the PBP2a(A77T) population versus the wild type (Fig. 4B). Scanning electron microscopy (SEM) also revealed that the poles of cells producing PBP2a(A77T) were rounded relative to the wild type and often lacked the characteristic pointed shape (Fig. 4C). Muropeptide analysis revealed the increased presence of higher-order crosslinked PG in the cells producing PBP2a(A77T), suggesting the production of an increased amount of multi-layered PG (Fig. 4D; Fig. S4B). However, transmission electron microscopy (TEM) did not detect any appreciable thickening of the PG layer in cells with the altered synthase (Fig. 4C). Overall, these results suggest that proper control of PBP2a function is required to maintain normal morphology in *Sp* cells and that MacP is likely to function at least in part via the stimulation of the PG polymerase activity of its partner synthase.

## DISCUSSION

Here, we investigated the regulation of PBP2a, an aPBP-type PG synthase from the Gram-positive pathogen *Sp*. PBP2a was previously shown to require its partner protein MacP for function, but the reason for the MacP requirement remained unclear (24). We identified amino acid substitutions in PBP2a that could bypass MacP function and mapped near the PGTase domain in a high-confidence AlphaFold model of the PBP2a structure (31, 32). Importantly, two of these PBP2a variants with MacP-bypass substitutions were found to have elevated PG polymerase activity in a purified system. Our results therefore suggest that MacP functions as a PGTase activator for PBP2a in *Sp* cells.

**Fig 5:**
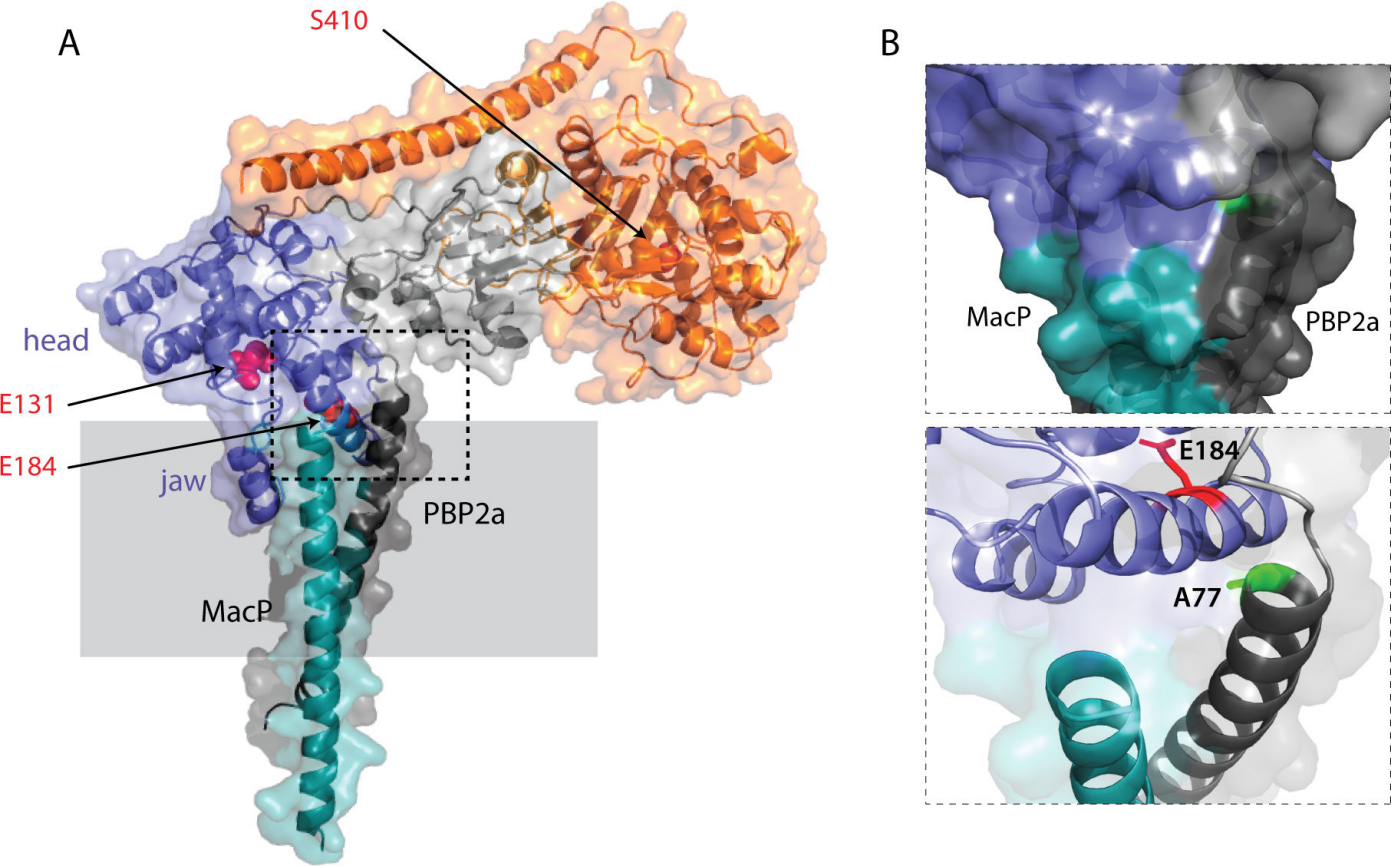
AlphaFold model of the complex between PBP2a and the transmembrane segment of MacP. (**A**) PBP2a is predicted to interact with the transmembrane segment of MacP. The TM segment of MacP is shown in cyan. The N-terminal cytoplasmic region and TM segment of PBP2a are shown in dark-gray; the GT domain (blue) and TP domain (orange) are flanked by the linker region (light-gray). Residues important for catalytic activity are highlighted in red. (**B**) Zoom in on the boxed region highlighting the interaction between the C-terminus of MacP and the lower lobe of the GT domain. The residue (**A77**) in PBP2a that, when mutated to T, I, P, or V, can bypass MacP is shown in green.

The location of amino acid substitutions conferring the MacP-bypass phenotype suggests a mechanism for how MacP activates PBP2a. In the solved structures of PGTases from aPBPs, the enzymatic domain consists of two subdomains called the “head” (upper lobe) and “jaw” (lower lobe). For both *E. coli* PBP1b and *S. aureus* PBP2, the jaw subdomain has been found to adopt different conformations when comparing the apo structure with those solved in the presence of the substrate-mimicking antibiotic moenomycin (25, 28–30). This subdomain is therefore thought to be conformationally flexible, with changes in this region potentially involved in controlling the activity of the enzyme. The jaw domain packs against the TM helix of the aPBP, and this interface is where the amino acid substitutions that activate *Sp* PBP2a are located. Furthermore, in an AlphaFold-predicted structure of the PBP2a-MacP complex, the extended TM helix of MacP interacts with the TM helix of PBP2a near its junction with the jaw of the PGTase domain (Fig. 5A and B; Fig. S5). We therefore propose that MacP promotes PBP2a activity by modulating the conformation of the jaw domain of PBP2a to activate PG polymerization.

In addition to changes at the interface between the TM and PGTase domains, amino acid substitutions in PBP2a present near the linker domain between the PGTase and TPase domains were also associated with the MacP-bypass phenotype. These substitutions are reminiscent of those in aPBPs from Gram-negative bacteria that activate their PGTase activity and allow them to function in the absence of their cognate outer membrane lipoprotein activators (15, 17). Notably, in addition to changes in the jaw domain, structures of the aPBPs from *E. coli* and *S. aureus* have also revealed flexibility in the orientation of the TPase and linker domains relative to the PGTase domain (28, 29, 36). Given that changes in the linker domain activate aPBPs from both Gram-positive and Gram-negative organisms, the interdomain flexibility observed in the structures is likely also involved in the regulation of the aPBPs. Although additional structural studies of activated variants of the aPBPs or aPBPs in complex with their cognate co-factors will be required to determine the detailed activation mechanism, the combination of genetic results from the different organisms suggests that distinct sets of co-factors could be inducing similar changes in their cognate PG synthases to stimulate their activity. In the case of the Gram-negative enzymes, the binding of the lipoproteins to accessory domains located near the interdomain linker may induce changes that are ultimately transduced to the jaw domain to activate PG polymerization. For MacP, the sequence may be reversed, with changes induced in the jaw domain of the PGTase by the TM helix of MacP also influencing the relative orientation between the PGTase and TPase domains to promote effective PG biogenesis.

In Gram-negative bacteria, the outer membrane localization of the lipoprotein activators is thought to play a role in controlling their access to their cognate aPBP. Areas, where the PG layer is less dense or gapped, are likely to be more favorable for the formation of a transenvelope complex between a lipoprotein activator and its cognate aPBP, thus directing aPBP activity to areas of the PG matrix in need of fortification (11). By contrast, in *Sp* cells, only a small portion of the MacP activator is extracytoplasmic. Its largest domain is cytoplasmic, which facilitates the regulation of MacP by the cell cycle kinase StkP (26, 37). Phosphorylation of MacP by this kinase is required for MacP to promote PBP2a activity but not for the MacP-PBP2a interaction (24). It currently remains unclear how phosphorylation of MacP in the cytoplasmic domain alters its ability to activate PBP2a. Nevertheless, proper cell cycle control of PBP2a appears to be critical because the PBP2a(A77T) variant capable of functioning without MacP results in aberrantly shaped poles and a wide distribution of cell sizes. Given the diversity of cell shapes, envelope architectures, and growth modes among bacteria, many different types of aPBP activators are likely to exist in nature that vary in their domain topology to provide additional levels of control. However, this work suggests that the underlying regulatory mechanism governing the aPBPs in each case may be similar.

## MATERIALS AND METHODS

### General methods

All *Sp* strains are derived from *S. pneumoniae* D39 ∆*cps* (38). Liquid cultures were grown in THY medium (Becton Dickinson) at 37°C in 5% CO_2_. For strain construction and spot dilutions, strains were grown on commercial Tryptic Soy Agar (TSA) with 5% sheep blood (TSAII 5% SB; Becton Dickinson) with a 5-mL overlay of 1% Nutrient Broth agar containing additives or TSA supplemented with 5% defibrinated sheep blood and additives. *Sp* strains were made competent using exponentially growing cells in THY that were back-diluted to an OD_600_ of 0.03 in THY supplement with 500 pg/mL competence-stimulating peptide 1 (CSP-1), 0.2% BSA, and 1 mM CaCl_2_. Cells were transformed with 100–200 ng of gDNA, plasmid DNA, or purified PCR product and selected on solid medium supplemented with the appropriate additives. Antibiotic concentrations used for selection were chloramphenicol 5 µg/mL, kanamycin 250 µg/mL, tetracycline 1.5 µg/mL, gentamycin 40 µg/mL, spectinomycin 100 µg/mL, and erythromycin 0.2 µg/mL. Cloning was performed in *E.coli* strain DH5α. Cells were selected on chloramphenicol 25 µg/mL, ampicillin 100 µg/mL, or kanamycin 40 µg/mL. All strains, plasmids, and oligonucleotides used in this study are listed in Tables S2–S5. The data in all figures are representative of one of three biological replicates except the SEM and TEM images.

### Viability assays

Cells were grown at 37°C in THY supplemented with 200 µM ZnCl_2_ until an OD_600_ of 0.3–0.5. Cultures were washed twice with THY lacking ZnCl_2_ and diluted to OD_600_ of 0.1- and 10-fold serially diluted. Aliquots (3 µL) of each dilution were then spotted on blood agar plates with and without 200 µM ZnCl_2_ as indicated.

### *pbp2a* mutant library construction

The *pbp2A* open reading frame in plasmid pCCM60 was PCR amplified using primers 417 and 418 using the error-prone polymerase Pfu(D473G) (36). The PCR product was cut with XbaI and SphI, purified from a 1% agarose gel, and subcloned into pCCM60 cut with XbaI and SphI. After transformation into *E. coli*, 10 transformants were sequenced, and the mutation rate was found to be ~1.5 mutations/kb. Approximately 250,000 transformants were pooled, and the plasmid library purified. The plasmid library was then transformed into *Sp* strain SP1047.

### Epifluorescence microscopy

Cells were grown to an OD_600_ between 0.3 and 0.5, concentrated 20 times, and spotted on a 2% agarose pad with THY or 1× PBS (phosphate bufered saline). All samples were imaged on a Nikon Ti-E inverted widefield microscope. Images were acquired using a Plan Apo 100×/1.40 Oil Ph3 DM objective lens with Cargille Type 37 immersion oil. Fluorescence was excited using a Lumencore SpectraX LED light engine and filtered using CFP (49001) and mCherry (49008) filter sets. Images were recorded on an Andor Zyla 4.2 Plus sCMOS camera (65-nm pixel size) using Nikon Elements (v5.10) acquisition software. Images were converted to tiff format using NIS-Element Viewer 4.50 or Fiji (39) prior to analysis with Metamorph software version 7.7.0.0. Cell width and length were determined from phase contrast images using MicrobeJ (40), ImageJ2 version 2.3.0/1.53 f, and MicrobeJ version 13 /m/16.

### Electron microscopy

Cells were grown in THY to an OD_600_ of 0.3, concentrated 20 times, and fixed with 2.5% glutaraldehyde and 0.125% Safranin O and Alcian blue overnight at 4°C for scanning electron microscopy or 0.1% osmium tetroxide (OSO_4_) for 1 hour followed by overnight fixation in 1% OSO_4_ at 4°C for transmission electron microscopy. Thermal plastic slides used for SEM were cleaned with methanol and coated with 0.1% poly-L-lysine. SEM imaging was performed by William Fowle at Northeastern University. TEM imaging was performed in the HMS Electron Microscopy Facility.

### Immunoblot analysis

Immunoblots were performed on membrane protein preparations using polyclonal anti-PBP2a antiserum generated in rabbit. Briefly, 25 mL of mid-exponential phase (OD_600_ = 0.3–0.5) cultures were normalized to an OD_600_ of 0.3 and harvested. Cell pellets were frozen, then resuspended in 500 µL lysis buffer [1× PBS, 1 mg/mL lysozyme, 25 units mutanolysin (Sigma), and 10 µg/mL DNaseI (NEB)], and incubated at 37°C for 1 hour. Samples were then sonicated 3 × 30 s (1 s pulse, 3 s rest, amplitude 25%) on ice. The lysate was centrifuged at 5,000 × *g* for 2 min to pellet unbroken cells. The supernatant was subjected to ultracentrifugation at 200,000 × *g* for 1 hour at 4°C. Membrane pellets were resuspended in 50 µL 2× PBS, and then, 50 µL of 2× SDS sample buffer with 10% β-mercaptoethanol was added. Membrane proteins were resolved by SDS-PAGE and transferred to PVDF membrane by semi-dry transfer. The membrane was blocked with 5% milk in 1× PBS containing 0.05% Tween 20 (PBST). The membrane was probed with anti-PBP2a antiserum diluted 1:10,000 with 3% BSA in PBST. Primary antibodies were detected with goat anti-rabbit IgG conjugated to horseradish peroxidase (Bio-Rad) used at a 1:3,000 dilution. Secondary antibodies were detected by enhanced chemiluminescence (Pierce SuperSignal) on the ChemiDoc Imaging System (Bio-Rad). Anti-PBP2a antiserum was generated using purified recombinant His-PBP2a, using a standard protocol (Covance).

### In-gel detection of HALO-PBP2a

Exponentially growing *Sp* cultures were normalized to an OD_600_ of 0.5, and 1 mL was withdrawn and incubated with 50 nM Janelia Fluor 549 (Promega) for 15 min at 37 °C with 5% CO_2_. Cells were pelleted and resuspended in 25 µL of 2× PBS with 1× complete protease inhibitors (complete EDTA free—Roche); then, 25 µL 2× SDS sample buffer with 10% β-mercaptoethanol was added. Proteins were resolved by SDS-PAGE, and gels were directly scanned using an Amersham Typhoon 5.

### Purification of *His-PBP2a* variants

*E. coli* Rosetta2 (DE3 pLysS) was transformed with pCCM97 [*His*-*pbp2a*(A77T)], pCCM98 [*His-pbp2a*(S410A)], pCCM99 [*His-pbp2a*(E131A)], or pMFS8 [*His-pbp2a*(WT)]. Fresh transformants were precultured in terrific broth (TB) supplemented with Kan (25 µg/mL) and Cm (25 µg/mL). Cultures were grown at 37°C to an OD_600_ of 0.4 and then used to inoculate 1 L of TB supplemented with antibiotics at an OD_600_ of 0.01. The cultures were grown at 37°C to an OD_600_ of 0.4, at which time they were cooled to room temperature (RT). IPTG (isopropyl thiogalactopyranoside) was added to 1.5 mM final concentration, and the cultures were grown overnight at 18°C. Cells were harvested by centrifugation at 4,667 × *g* for 15 min, and pellets were weighed and frozen. Cell pellets were resuspended in 10× the pellet volume with buffer A (20 mM Tris-HCl pH 7.4, 150 mM NaCl, 10% glycerol, 2× complete protease inhibitors), sonicated, and further lysed by three passes through a cell disruptor at 25,000 PSI at 4°C (Constant Systems). The lysates were subjected to ultracentrifugation at 200,000 × *g* for 1 hour, at 4°C. The membrane pellets were dounce homogenized in 30 mL of buffer B (20 mM Tris-HCl at pH 7.4, 150 mM NaCl, 10% glycerol, 1% wt/vol CHAPS). The homogenate was rolled for 1 hour at 4°C, followed by ultracentrifugation at 200,000 × g for 1 hour, at 4°C. The detergent-solubilized membrane proteins (S100) were passed through a 0.22 PES filter prior to purification on the AKTA purifier system using a HisTrap column (1 mL, GE). After loading and washing with buffer C (20 mM Tris-HCl at pH 7.4, 500 mM NaCl, 10% glycerol, 0.1% reduced Triton-X-100), the sample was eluted with a gradient of buffer D (20 mM Tris-HCl at pH 7.4, 500 mM NaCl, 10% glycerol, 0.1% reduced Triton-X-100, 500 mM imidazole). Peak fractions were pooled and dialyzed overnight at 4°C in buffer C. The proteins were analyzed for purity by SDS-PAGE using 4%–20% polyacrylamide gels stained with InstantBlue (Expedeon).

### *In vitro* glycosyltransferase activity assay

Glycosyltransferase activity assays were performed using a protocol adapted from (35). Briefly, a 10× MTG buffer [125 mM HEPES (pH7.5), 20 mM MnCl_2_, 2.5 mM Tween 80] was prepared. Master mixes of the reactions were prepared such that the final conditions were as follows: 0.1 µM purified His-PBP2a variant, 1× MTG buffer, 2% DMSO, 10 µM lipid II purified from *E. faecalis* as described in (33), and 200 µM cephalexin to block transpeptidase activity. Cephalexin was not included in the experiments presented in Fig. S4.

Reactions were initiated by the addition of lipid II and immediately placed at 25°C. Aliquots (10 µL) of the reaction mixture were withdrawn at the indicated times and stopped by heat denaturation for 5 min at 95°C. Following inactivation, recombinant *S. aureus* PBP4 (5 µM final concentration) (34) and biotinylated D-lysine (200 µM final concentration; Sigma) were added, and the samples were incubated for 1 hour at 37°C. Reactions were quenched by the addition of 15 µL of 2× SDS sample buffer with 10% β-mercaptoethanol, and 5 µL of the reaction was resolved by SDS-PAGE on a 4%–20% acrylamide gel for 80 min at 35 mA. The samples were transferred to a PVDF membrane and washed with TBS (Tris-buffered saline). The membrane was incubated in 0.4% paraformaldehyde for 1 hour, washed with 1× PBS, and then blocked for 1 hour in SuperBlock (Thermo). The membrane was then incubated in SuperBlock with IRDye 800CW streptavidin (diluted 1:5,000) for 1 hour at room temperature. Membranes were washed three times in TBS/Tween 20 and once in TBS and imaged using an Odyssey CLx System (LI-COR Biosciences).

### Muropeptide analysis

Lyophilized, *Sp* cells were resuspended in 48% (vol/vol) hydrofluoric acid and incubated at 4°C for 48 hours with mild stirring. Sacculi were then pelleted, washed several times in water, once in Tris-HCl (pH 6.8), and then resuspended in muramidase buffer (50 mM phosphate buffer, pH 4.9) and digested with mutanolysin (Sigma) overnight at 37°C. Muropeptides were then adjusted to pH 9.0 using borate buffer, reduced with NaBH4, and pH adjusted to ~2.0 with 25% (vol/v) orthophosphoric acid.

Muropeptide composition was determined on an Ultra-Performance Liquid Chromatography System interfaced with a Xevo G2/XS Q-TOF mass spectrometer (Waters Corp.).Chromatographic separation was achieved using an ACQUITY UPLC BEH C18 Column (2.1 mm × 150 mm, 1.7 µm pore size) heated to 45°C. Formic acid (0.1%) in Milli-Q water (buffer A) and formic acid (0.1%) in acetonitrile (buffer B) were used as eluents. The gradient of buffer B was set as follows: 0–3 min 5%, 3–6 min 5%–6.8%, 6–7.5 min 6.8%–9%, 7.5–9 min 9%–14%, 9–11 min 14%–20%, 11–12 min hold at 20% with a flow rate of 0.25 mL/min; 12–12.10 min 20%–90%, 12.1–13.5 min hold at 90%, 13.5–13.6 min 90%–2%, 13.6–16 min hold at 2% with a flow rate of 0.3 mL/min; and then 16–18 min hold at 2% with a flow rate of 0.25 mL/min. Chromatograms were recorded at 204 nm. The QTOF-MS instrument was operated in positive ionization mode.

Relative total PG amounts were calculated by comparison of the total intensities of the chromatograms (total area) from three biological replicates extracted with the same volumes. Muropeptide identity was confirmed by MS/MS analysis using a Xevo G2-XS QTOF system (Waters Corp.). Quantification of muropeptides was based on their relative abundances (relative area of the corresponding peak) normalized to their molar ratio. Analyses were performed in biological triplicates, and means were compared with unpaired *t*-tests.

### Structural predictions

Structure prediction of PBP2a and MacP/Pbp2a complex were run using ColabFold (AlphaFold2_mmseqs2) (31, 32) with the following parameters: num_relax:0; template_mode: none; msa_mode: mmseq2_uniref_env; and pair _mode: unpaired_paired, recycling 3. The first 63 amino acids of MacP corresponding to the cytoplasmic domain were not included in the analysis. Model visualization and figure preparation were performed using PyMol Molecular Graphics System version 2.5.4 (Schrödinger, L. & DeLano, W., 2020. PyMOL, available at: http://www.pymol.org/pymol).

## SUPPLEMENTAL MATERIAL

Supplemental material mbio.02390-23-s0001.pdf Fig. S1 to S5 and Tables S1 to S5.

ASM does not own the copyrights to Supplemental Material that may be linked to, or accessed through, an article. The authors have granted ASM a non-exclusive, world-wide license to publish the Supplemental Material files. Please contact the corresponding author directly for reuse.
