# Supplementary material for: MacP bypass variants of Streptococcus pneumoniae PBP2a suggest a conserved mechanism for the activation of bifunctional cell wall synthases
Source: mBio. 2023 Oct 17;14(6):e02390-23. doi: 10.1128/mbio.02390-23 (PMC10746261; doi:10.1128/mbio.02390-23)
Supplement: Supplemental material — Fig. S1 to S5 and Tables S1 to S5. [file mbio.02390-23-s0001.pdf]

Supplementary Information For:

MacP bypass variants of *Streptococcus pneumoniae* PBP2a suggest a conserved mechanism for the activation of bifunctional cell wall synthases.

Authors: Caroline Midonet[1], Sean Bisset[3], Irina Shlosman[4], and Felipe Cava[5,6], David Z. Rudner[1*], and Thomas G. Bernhardt[1,2*].

Affiliations:
[1]Department of Microbiology
Harvard Medical School, Blavatnik Institute
Boston, Massachusetts 02115, USA

[2]Howard Hughes Medical Institute

[3]Department of Molecular Biology,
Umeå University
SE-901 87, Umeå, Sweden.

[4]Department of Biological Chemistry and Molecular Pharmacology
Harvard Medical School, Blavatnik Institute
Boston, Massachusetts 02115, USA

[5]Laboratory for Molecular Infection Medicine Sweden (MIMS),
Umeå Center for Microbial Research (UCMR).

[6]Science for Life Laboratory (SciLifeLab).
Department of Molecular Biology,
Umeå University, Sweden.

*To whom correspondence should be addressed
Thomas G. Bernhardt
Harvard Medical School
Department of Microbiology
Boston, Massachusetts 02115, USA
e-mail: thomas_bernhardt@hms.harvard.edu

David Z. Rudner
Harvard Medical School
Department of Microbiology
Boston, Massachusetts 02115, USA
e-mail: david_rudner@hms.harvard.edu

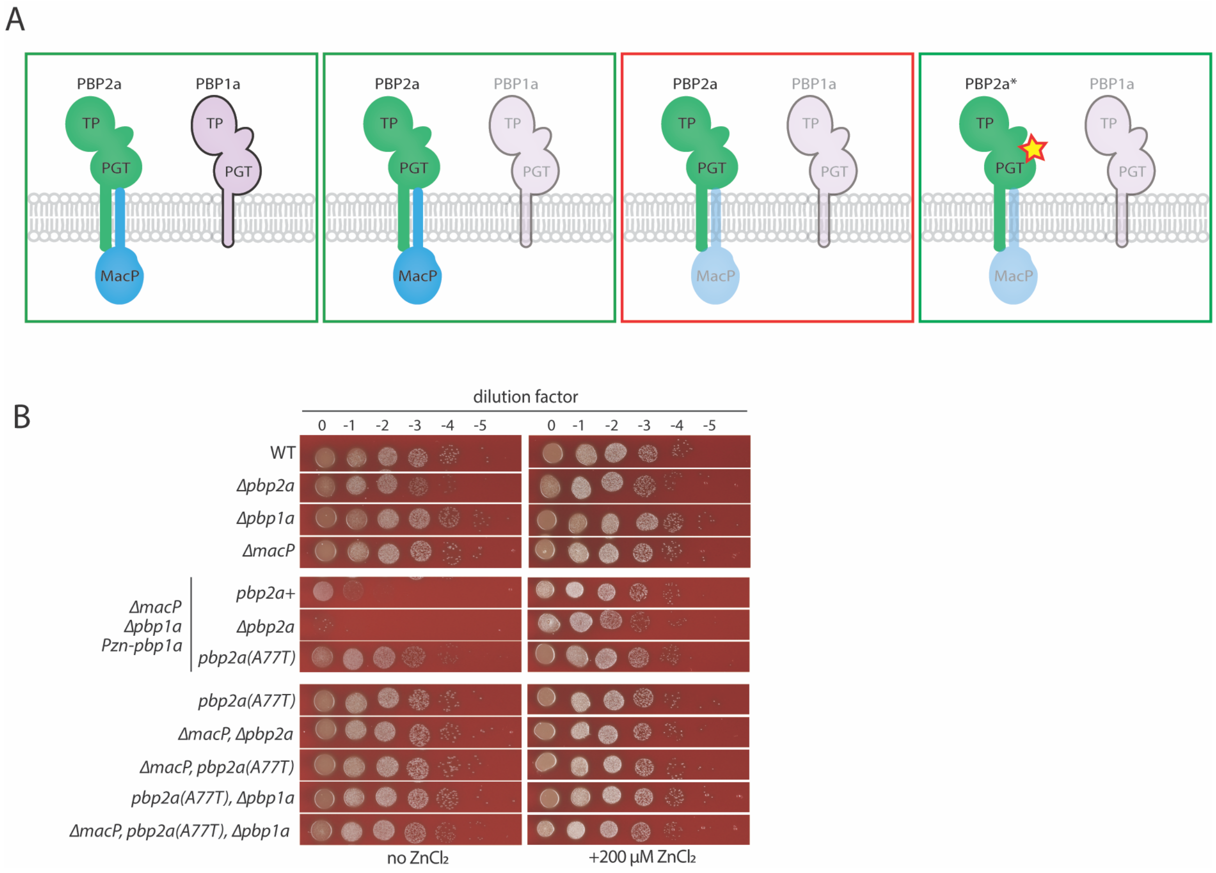

Figure S1. Schematic representation of the selection for PBP2a mutants that bypass the requirement for MacP. (A) Boxes depict viable (green) and non-viable (red) strains. PPB1a depletion and *macP* deletion are represented as translucent shapes. PBP2a mutants that bypass the requirement for MacP are shown with a yellow star. **(B)** Representative spot dilutions of the indicated strains on blood agar plates in the absence and presence of 200 µM $ZnCl_2$. The data in the figure are from one of three biological replicates.

A

PBP2a mutants tested for growth in Δ*macP*, Δ*pbp1a*, *Pzn-pbp1A* background														
A75	V76	A77	K78	S79	T80	D84	K90	R92	D97	R98	E99	E100	K101
A75T	**V76T**	A77C	K78A	S79A	T80A	**D84K**	K90D	**R92D**	D97K	R98D	E99K	E100K	K101D
		A77D	K78D	S79V	T80V			**R92E**			E99R	E100R	
		A77E											
		A77F											
		A77G											
		A77H											
		**A77I**											
		A77K											
		A77L											
		A77M											
		A77N											
		**A77P**											
		A77Q											
		A77R											
		A77S											
		**A77T**											
		**A77V**											
		A77W											
		A77Y											

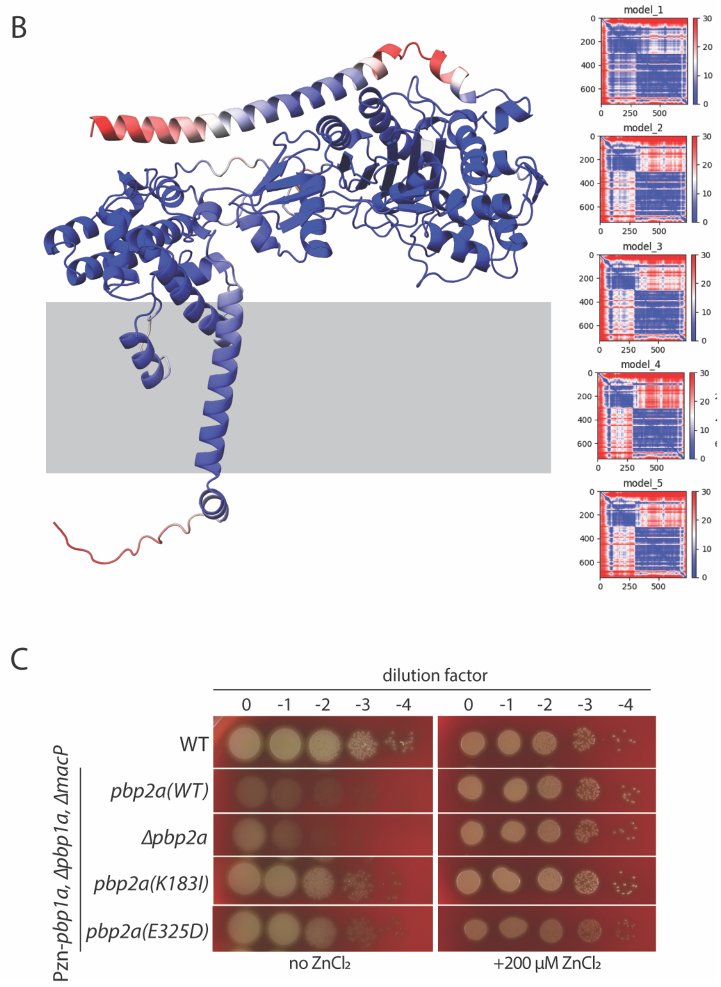

Figure S2. PBP2a variants tested for the ability to bypass MacP
(A) Table of the PBP2a variants generated by site-directed mutagenesis and tested for the ability to support growth of a Δ*macP* mutant upon depletion of PBP1a. Each mutant was tested for growth in strain Sp1047 (*Δpbp1a, ΔmacP, Pzn-pbp1a, Δpbp2a*) by introducing the indicated *pbp2a* allele. Mutants that were unable to sustain growth when PBP1a was depleted are shown in black. Mutants that could bypass MacP are shown in

green. **(B**) Structural model of PBP2a with predicted local distance difference test (pLDDT) for all residues in the predicted structure. Low (red) to high (blue) confidence is indicated. The predicted alignment error (pAE) plots for the top 5 models generated. **(C)** Representative spot dilutions of the indicated strains on blood agar plates in the absence and presence of $ZnCl_2$. Mutants K183I and E325D also bypass MacP requirements. Data in panel C are from one of three biological replicates.

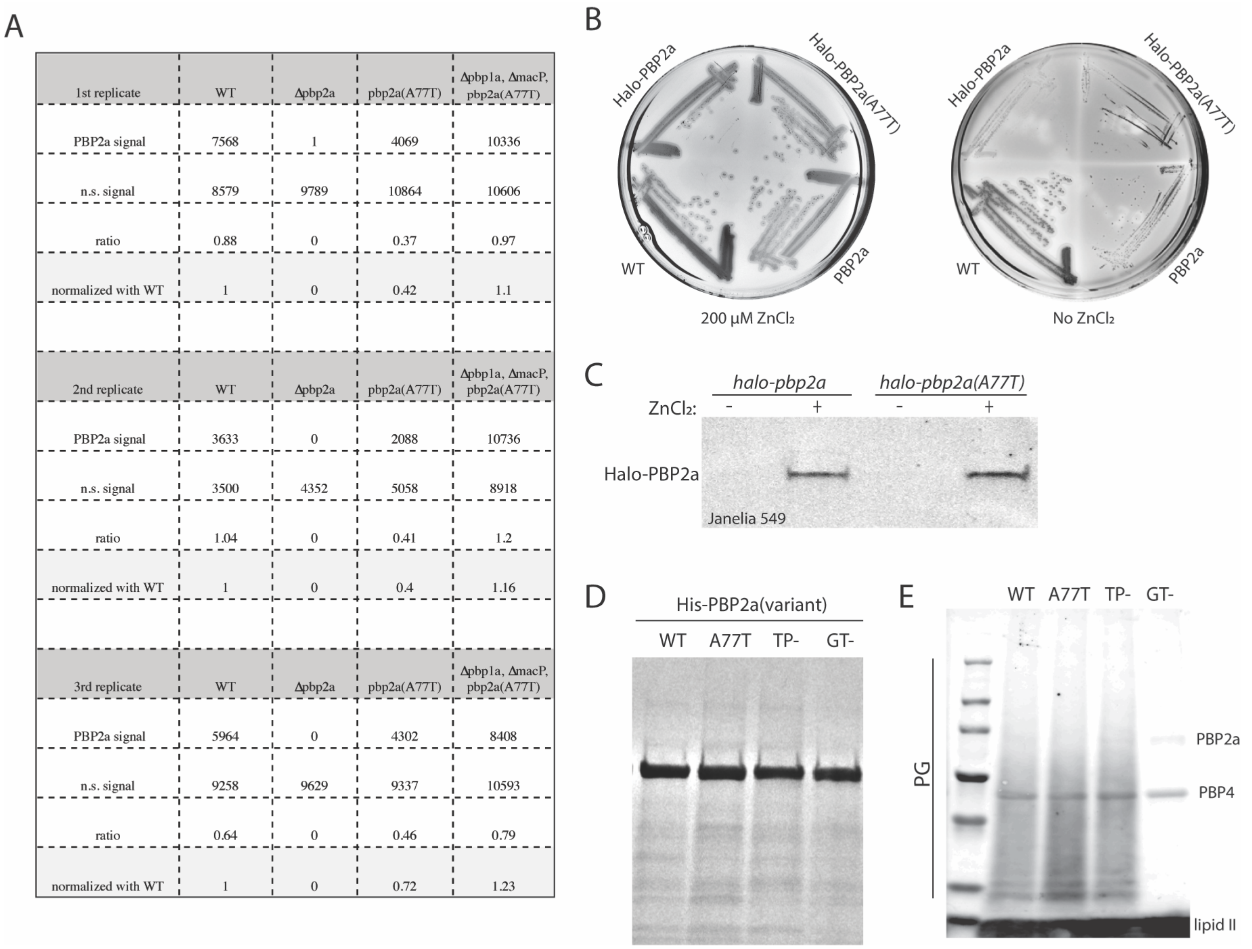

1st replicate	WT	Δpbp2a	pbp2a(A77T)	Δpbp1a, ΔmacP, pbp2a(A77T)
PBP2a signal	7568	1	4069	10336
n.s. signal	8579	9789	10864	10606
ratio	0.88	0	0.37	0.97
normalized with WT	1	0	0.42	1.1
2nd replicate	WT	Δpbp2a	pbp2a(A77T)	Δpbp1a, ΔmacP, pbp2a(A77T)
PBP2a signal	3633	0	2088	10736
n.s. signal	3500	4352	5058	8918
ratio	1.04	0	0.41	1.2
normalized with WT	1	0	0.4	1.16
3rd replicate	WT	Δpbp2a	pbp2a(A77T)	Δpbp1a, ΔmacP, pbp2a(A77T)
PBP2a signal	5964	0	4302	8408
n.s. signal	9258	9629	9337	10593
ratio	0.64	0	0.46	0.79
normalized with WT	1	0	0.72	1.23

Figure S3. Analysis of PBP2A *in vivo* and *in vitro*.

(A) Table documenting the relative amounts of PBP2a in the indicated strains. Immunoblots from three biological replicates were analyzed, and the intensities (arbitrary units) of PBP2a and the non-specific (n.s.) bands were quantified using ImageJ. No significant enrichment was observed for Δ*pbp1a,* Δ*macP,* or *pbp2a*(A77T) strains. However, PBP2a(A77T) levels were reduced in strains harboring MacP and PBP1A. **(B)** Representative streaks of the indicated strains on blood agar plates in the absence and presence of 200 µM $ZnCl_2$. With the exception of wild-type (WT), the other strains lack *pbp1a* and *pbp2a* and harbor the Pzn promoter fused to *pbp2a*, *halo-pbp2a,* or *halo-pbp2a(A77T)*. Expression of Halo-PBP2a variants supports growth in strains lacking *pbp1a* and *pbp2a*. **(C)** Representative image of SDS-PAGE gel in which cells harboring Halo-PBP2a variants were labeled with Janelia fluor 549 and lysates resolved by SDS-PAGE and imaged directly using the Typhoon FLA9500. Cells grown in the absence or presence of 200 µM $ZnCl_2$ were incubated with the Janelia fluor 549 (50nM for 15 min) prior to lysis. **(D)** InstantBlue-stained gel of the purified His-PBP2a variants used in the *in vitro* glycosyltransferase assay. The catalytic mutants in the transpeptidase domain (S410A) and the glycosyltransferase domain (E131A) are labeled TP- and GT-, respectively. **(E)** Representative blot of glycan strand polymerization assay using purified proteins and lipid II. The reaction products and remaining substrate were labeled with

biotinylated D-lysine using *S. aureus* PBP4, resolved by SDS-PAGE, transferred to PVDF, and detected by IRDye 800CW Streptavidin. Free lipid II and glycan strands (PG) are indicated. Some PBP4 and PBP2a become biotinylated in the labeling reaction and are detected by IRDye 800CW Streptavidin. Timepoint: 2 min. A final concentration of 0.5μM of PBP2a variants and no cephalexin were used for this specific control.

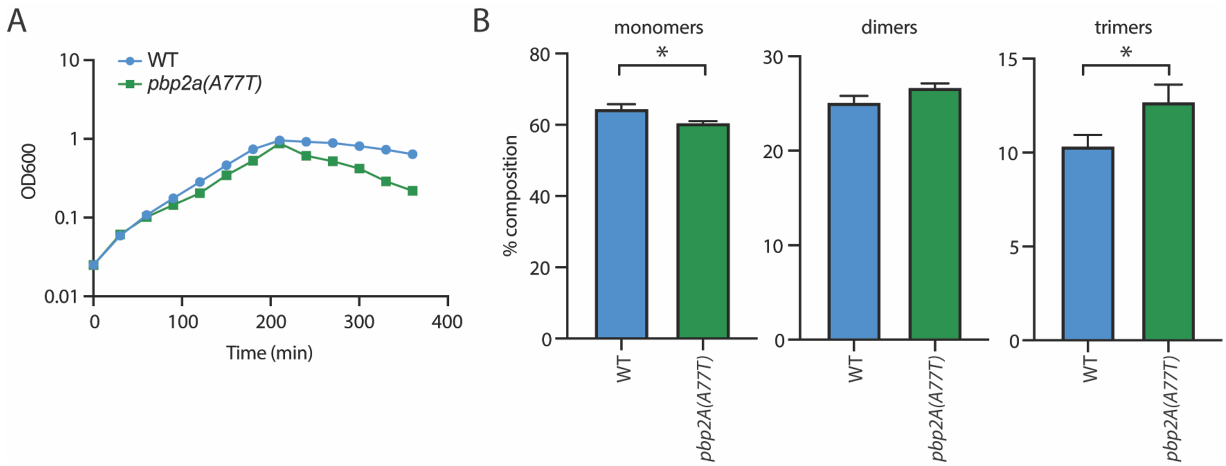

Figure S4 Analyses of strains harboring *pbp2A*(WT) and *pbp2A*(A77T)
(A) Growth curve in THY medium of the indicated strains. **(B)** Bar graph shows the percentage of cell wall crosslinking in the indicated strains. The difference is statistically significant (*) with a *p-value* < *0.05 (p-value*= 0.018 for the monomers plot; *p-value*= 0.048 for the trimers plot). Monomers, dimers, and trimers refer to uncrosslinked, singly crosslinked, or doubly crosslinked muropeptides detected in the muropeptide analysis of *Sp* peptidoglycan.

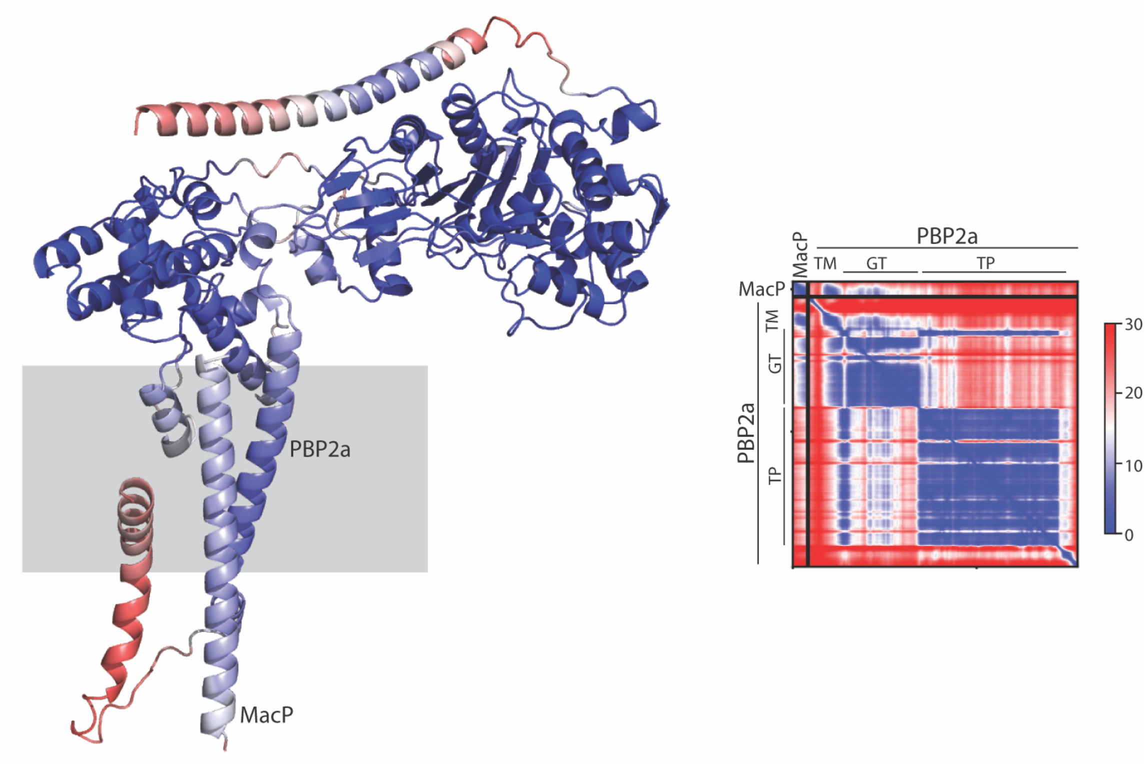

Figure S5: Predicted local difference test and alignment error for the AlphaFold-predicted complex of PBP2a and the transmembrane segment of MacP. Structural model of the PBP2a and the transmembrane segment of MacP with predicted local distance difference tests (pLDDT) per position mapped onto predicted complex (left). Red-white-blue colors indicate low to high confidence. The predicted alignment error in Å of all residues against all residues for the top-ranked model is shown on the right. Given that the extreme N-terminus of PBP2a is unlikely to fold back into the membrane as predicted by AlphaFold, we eliminated this portion of the protein structure from the models shown in other figures for clarity.

Table S1

PBP2a suppressor number	number of mutations	change(s)
3a	4	A167V, G577A, T593T, A699V
29a	6	A105T, Y136Y, N331K, K392K, S404A, E705V
30a	2	A77T, P375S
39a	1	A77T
41a	2	A77T
44a	2	D84N, T571S
4b	1	E325D
9b	4	D84G, N342N, L477F, A719V
14b	2	A77T, P375S
17b	4	L73S, G385A, S535C, P653P
21b	1	A77T
4c	2	A77T, P375S
14c	2	A77T, P375S
16c	-	T571S, poor sequencing
56c	2	K183I, P673L

TableS1: Isolated variants of PBP2a that promote growth upon PBP1a depletion in absence of MacP. Red: A77T mutation with the highest occurrence; blue: substitutions also tested as a single change (D84K and K183I) in validation studies.

Table S2

WT	*D39 Δcps*	lab collection
6	*D39 Δcps, Δpbp2a::Erm*	Fenton A. et al. 2018
9	*D39 Δcps, Δpbp1a::Kan*	Fenton A. et al. 2016
30	*D39 Δcps, Δpbp1a::Kan, ΔbgaA::*Pzn-*pbp1a-Tet, Δpbp2a:Erm*	Fenton A. et al. 2018
36	*D39 Δcps, Δpbp1a::Kan, ΔbgaA::*Pzn-*pbp1a-Tet*	Fenton A. et al. 2018
94	*D39 Δcps, ΔmacP::Spec*	Fenton A. et al. 2018
106	*D39 Δcps, Δpbp1a::Kan, ΔmacP::Spec, ΔbgaA::pZn-pbp1a-Tet*	Fenton A. et al. 2018
1047	*D39 Δcps, Δpbp1A:kan, ΔbgaA::Pzn-pbp1A:tet, ΔmacP:Spec, Δpbp2a:erm*	This study
1064	*D39 Δcps, Δpbp1A:kanR, ΔbgaA::Pzn-pbp1A-tetR, ΔmacP:SpecR, pbp2a(A77T)-CmR*	This study
1074	*D39 Δcps, Δpbp2a::pbp2a(A77T)-Cm*	This study
1075	*D39 Δcps, Δpbp2a::Erm, ΔmacP::Spec*	This study
1082	*D39 Δcps, ΔmacP::Spec, Δpbp2a::pbp2a(A77T)-Cm*	This study
1083	*D39 Δcps, Δpbp2a::pbp2(A77T) CmR, ΔSPD_0876(macP)::SpecR*	This study
1086	*D39 Δcps, Δpbp1a::Kan, Δpbp2a::pbp2a(A77T)-Cm*	This study
1087	*D39 Δcps, Δpbp1a::Kan, ΔmacP::Spec, Δpbp2a::pbp2a(A77T)-Cm*	This study
1094	*D39 Δcps, 1526:PF6-optRBS-CFP:1527 KanR*	This study
1095	*D39 Δcps, 1526:PF6-optRBS-CFP:1527 KanR, pbp2a(A77T)-CmR*	This study
1115	*D39 Δcps, Δpbp1A::Kan, ΔbgaA::*Pzn-*pbp1A-TetR, ΔmacP::Spec, Δpbp2a::pbp2a(A77I)-CmR*	This study
1116	*D39 Δcps, Δpbp1A::Kan, ΔbgaA::*Pzn-*pbp1A-TetR, ΔmacP::Spec, Δpbp2a::pbp2a(A77V)-CmR*	This study
1117	*D39 Δcps, Δpbp1A::Kan, ΔbgaA::*Pzn-*pbp1A-TetR, ΔmacP::Spec, Δpbp2a::pbp2a(A77P)-CmR*	This study
1118	*D39 Δcps, Δpbp1A::Kan, ΔbgaA::*Pzn-*pbp1A-TetR, ΔmacP::Spec, Δpbp2a::pbp2a(V76T)-CmR*	This study
1119	*D39 Δcps, Δpbp1A::Kan, ΔbgaA::*Pzn-*pbp1A-TetR, ΔmacP::Spec, Δpbp2a::pbp2a(R92E)-CmR*	This study
1120	*D39 Δcps, Δpbp1A::Kan, bgaA::*Pzn-*pbp1A-TetR, ΔmacP::Spec, Δpbp2a::pbp2a(D84K)-CmR*	This study
1121	*D39 Δcps, Δpbp1A::Kan, ΔbgaA::*Pzn-*pbp1A-TetR, ΔmacP::Spec, Δpbp2a::pbp2a(R92D)-CmR*	This study
1124	*D39 Δcps, Δpbp2a::erm, ΔbgaA::Pzn-HALO-pbp2a-tetR*	This study
1146	*D39 Δcps, Δpbp2a::erm, ΔbgaA::Pzn-HALO-pbp2a-tetR, Δpbp1a::kanR*	This study
1148	*D39 Δcps, Δpbp2a::erm, ΔbgaA::Pzn-HALO-pbp2a(A77T)-tetR*	This study
1150	*D39 Δcps, Δpbp2a::erm, ΔbgaA::Pzn-HALO-pbp2a(A77T)-tetR, Δpbp1a::kanR*	This study
1158	*D39 Δcps, Δpbp1A::Kan, ΔbgaA::*Pzn-*pbp1A-TetR, ΔmacP::Spec, Δpbp2a::pbp2a(E325D)-CmR*	This study
1160	*D39 Δcps, Δpbp1A::Kan, ΔbgaA::*Pzn-*pbp1A-TetR, ΔmacP::Spec, Δpbp2a::pbp2a(K183I)-CmR*	This study

Table S2: *Sp* strains used for this study.

Table S3

plasmid	genotype	Reference
pCCM60	ori pACYC, Δpbp2a: Pnative-pbp2a-cat, bla	This study
pCCM64	ori pACYC, Δpbp2a: Pnative-pbp2a(A77T)-cat, bla	This study
pMFS8	ori pBR/colE1, kanR, lacIq, PT7:His-*pbp2a*	This study
pCCM97	ori pBR/colE1, kanR, lacIq, PT7:His-*pbp2a*(A77T)	This study
pCCM98	ori pBR/colE1, kanR, lacIq, PT7:His-*pbp2a_TP- (*S410A)	This study
pCCM99	ori pBR/colE1, kanR, lacIq, PT7:His-*pbp2a*_GT- (E131A)	This study
pCCM114	ori pBR/colE1, kanR, lacIq, PT7:His-*pbp2a(D84K)*	This study
Genscript synthetized PBP2a(A77I)	ori pACYC, Δpbp2a: Pnative-*pbp2a(A77I*)-cat, bla	This study
Genscript synthetized PBP2a(A77V)	ori pACYC, Δpbp2a: Pnative-*pbp2a(A77V*)-cat, bla	This study
Genscript synthetized PBP2a(A77P)	ori pACYC, Δpbp2a: Pnative-*pbp2a(A77P*)-cat, bla	This study
Genscript synthetized PBP2a(V76T)	ori pACYC, Δpbp2a: Pnative-*pbp2a(V76T)*-cat, bla	This study
Genscript synthetized PBP2a(R92E)	ori pACYC, Δpbp2a: Pnative-*pbp2a(R92E*)-cat, bla	This study
Genscript synthetized PBP2a(D84K)	ori pACYC, Δpbp2a: Pnative-*pbp2a(D84K)*-cat, bla	This study
Genscript synthetized PBP2a(R92D)	ori pACYC, Δpbp2a: Pnative-*pbp2a*(R92D)-cat, bla	This study
Genscript synthetized PBP2a(A75T)	ori pACYC, Δpbp2a: Pnative-*pbp2a*(A75T)-cat, bla	This study
Genscript synthetized PBP2a(A77C)	ori pACYC, Δpbp2a: Pnative-*pbp2a*(A77C)-cat, bla	This study
Genscript synthetized PBP2a(A77D)	ori pACYC, Δpbp2a: Pnative-*pbp2a*(A77D)-cat, bla	This study
Genscript synthetized PBP2a(A77E)	ori pACYC, Δpbp2a: Pnative-*pbp2a*(A77E)-cat, bla	This study
Genscript synthetized PBP2a(A77F)	ori pACYC, Δpbp2a: Pnative-*pbp2a*(A77F)-cat, bla	This study
Genscript synthetized PBP2a(A77G)	ori pACYC, Δpbp2a: Pnative-*pbp2a*(A77G)-cat, bla	This study
Genscript synthetized PBP2a(A77H)	ori pACYC, Δpbp2a: Pnative-*pbp2a*(A77H)-cat, bla	This study
Genscript synthetized PBP2a(A77K)	ori pACYC, Δpbp2a: Pnative-*pbp2a*(A77K)-cat, bla	This study
Genscript synthetized PBP2a(A77L)	ori pACYC, Δpbp2a: Pnative-*pbp2a*(A77L)-cat, bla	This study
Genscript synthetized PBP2a(A77M)	ori pACYC, Δpbp2a: Pnative-*pbp2a*(A77M)-cat, bla	This study
Genscript synthetized PBP2a(A77N)	ori pACYC, Δpbp2a: Pnative-*pbp2a*(A77N)-cat, bla	This study
Genscript synthetized PBP2a(A77P)	ori pACYC, Δpbp2a: Pnative-*pbp2a*(A77P)-cat, bla	This study
Genscript synthetized PBP2a(A77Q)	ori pACYC, Δpbp2a: Pnative-*pbp2a*(A77Q)-cat, bla	This study

Genscript synthetized PBP2a(A77R)	ori pACYC, Δpbp2a: Pnative-*pbp2a*(A77R)-cat, bla	This study
Genscript synthetized PBP2a(A77S)	ori pACYC, Δpbp2a: Pnative-*pbp2a*(A77S)-cat, bla	This study
Genscript synthetized PBP2a(A77W)	ori pACYC, Δpbp2a: Pnative-*pbp2a*(A77W)-cat, bla	This study
Genscript synthetized PBP2a(A77Y)	ori pACYC, Δpbp2a: Pnative-*pbp2a*(A77Y)-cat, bla	This study
Genscript synthetized PBP2a(K78A)	ori pACYC, Δpbp2a: Pnative-*pbp2a*(K78A)-cat, bla	This study
Genscript synthetized PBP2a(K78D)	ori pACYC, Δpbp2a: Pnative-*pbp2a*(K78D)-cat, bla	This study
Genscript synthetized PBP2a(S79A)	ori pACYC, Δpbp2a: Pnative-*pbp2a*(S79A)-cat, bla	This study
Genscript synthetized PBP2a(S79A)	ori pACYC, Δpbp2a: Pnative-*pbp2a*(S79V)-cat, bla	This study
Genscript synthetized PBP2a(T80A)	ori pACYC, Δpbp2a: Pnative-*pbp2a*(T80A)-cat, bla	This study
Genscript synthetized PBP2a(T80V)	ori pACYC, Δpbp2a: Pnative-*pbp2a*(T80V)-cat, bla	This study
Genscript synthetized PBP2a(K90D)	ori pACYC, Δpbp2a: Pnative-*pbp2a*(K90D)-cat, bla	This study
Genscript synthetized PBP2a(D97K)	ori pACYC, Δpbp2a: Pnative-*pbp2a*(D97K)-cat, bla	This study
Genscript synthetized PBP2a(R98D)	ori pACYC, Δpbp2a: Pnative-*pbp2a*(R98D)-cat, bla	This study
Genscript synthetized PBP2a(E99K)	ori pACYC, Δpbp2a: Pnative-*pbp2a*(E99K)-cat, bla	This study
Genscript synthetized PBP2a(E99R)	ori pACYC, Δpbp2a: Pnative-*pbp2a*(E99R)-cat, bla	This study
Genscript synthetized PBP2a(E100K)	ori pACYC, Δpbp2a: Pnative-*pbp2a*(E100K)-cat, bla	This study
Genscript synthetized PBP2a(E100R)	ori pACYC, Δpbp2a: Pnative-*pbp2a*(E100R)-cat, bla	This study
Genscript synthetized PBP2a(K101D)	ori pACYC, Δpbp2a: Pnative-*pbp2a*(K101D)-cat, bla	This study
pCCM113	ori pACYC, Δpbp2a: Pnative-*pbp2a*(K183I)-cat, bla	This study
pGD159	pACYC, SPD_1526- PF6 promoter-SCFP3A-aphA-3, SPD_1527	This study

Table S3: Plasmids Table. Plasmids highlighted in grey were ordered and mutagenized by Genscript.

Table S4

Oligonu-cleotide	Sequence (5'->3')	use
45	GAGGGAGGAAAGGCAGGA	construction pCCM60
46	CGCCGTATCTGTGCTCTC	construction pCCM60
388	TCATGACAGATGAAGCTTGGCAGACAATTGACGGCTTGACG G	construction pCCM60
389	CCAAGCTTCATCTGTCATGACCATTTCTAC	construction pCCM60
390	GAGAGCACAGATACGGCGTCAAAGCCTAGCTTTCTTGTTATA ATGGATAAGATG	construction pCCM60
391	TCCTGCCTTTCCTCCCTCCGCATGCAAGCATCTTAGCGAAAT AGATTGACTATCG	construction pCCM60
392	ACTAGTGCAAAAGGTGCACACGAATCTGATG	construction pCCM60
393	CATCAGATTCGTGTGCACCTTTTGCACTAGTCAGGTGGCACT TTTCGGGG	construction pCCM60
394	AGGGATGAGGAGTTGTTCCTCAAG	sequencing pbp2a locus/plasmid
395	GTTCAGGTGGTGGCTCTACCATTAC	sequencing pbp2a locus/plasmid
396	GCAGAGGATGGAACGTTTGCTC	sequencing pbp2a locus/plasmid
397	TCTGTAGCTGACAAGATGACCAGTATGATG	sequencing pbp2a locus/plasmid
398	GCACTTTGATATCGACCCAAGTACCG	sequencing pbp2a locus/plasmid
399	GATTTTCGTTCTATCATGGAATACACAGCTTTC	sequencing pbp2a locus/plasmid
400	GATGATCTTCTTGAGATCGTTTTGGTCTG	sequencing pbp2a locus/plasmid
401	GATCGTTGTCAGAAGTAAGTTGGCCG	sequencing pbp2a locus/plasmid
417	TCCCTCCGCATGCAAGCATC	random mutagenesis pbp2a
418	CCATCAACTAGAGCCCAATATTCTCTAGAAATC	random mutagenesis pbp2a
441	GAATCTATTTGTTTGCTGTAACCAAGTCGACCAATGTCAATG	mutation pbp2a(A77T)
441	GAATCTATTTGTTTGCTGTAACCAAGTCGACCAATGTCAATG	construction pbp2a(A77T)
469	GTTGAGAGAAAAGCGATAGAATTTTTCCTTGCCTTAG	construction pbp2a(K183I)
522	CCAATCAAAGCGTAGTCCTGGTGCTACAATTAAGCCTTTAGTT GTTTATAC	mutation pbp2a_TP- (S410A)
523	AACTTGCAGAATGCTGTTATTGCGACAGCAGACCGTTCTTTC TATAAAAATGACGG	mutation pbp2a_GT- (E131A)
545	CAAGTCGACCAATGTCAATAAATTGCAAAATGCCTTGAAAACT CGG	construction pbp2a(D84K)

Table S4: Oligonucleotides/primers table

Table S5

strain	genotype	Reference
DH5α λpir	endA1 hsdR17 glnV44 (= supE44) thi-1 recA1, gyrA96 relA1 ϕ80dlacΔ(lacZ)M15 Δ(lacZYAargF), U169 zdg-232::Tn10 uidA::pir+	Gibco BRL
Rosetta2(DE3)	F- ompT hsdSB(rB- mB-) gal dcm (DE3), pRARE2 (CamR)	Novagen
MMH594	Enterococcus faecalis EnGen0310	Huycke et al. 1991

Table S5: Other bacterial strains. DH5α λpir and Rosetta2(D33) are E.*coli* strains respectively used for cloning and proteins expression. MMH594 is an Enterococcus *faecalis* strain used for purification of lipid II.
